# Correlation Analysis between Volatile Compounds and Quality Attributes in Pork Tenderloin in Response to Different Stir-Frying Processes

**DOI:** 10.3390/foods12234299

**Published:** 2023-11-28

**Authors:** Ziqiang Wang, Tianjie Nie, Huiying Zhang, Wenqian Wang, Haitao Chen, Shuqi Wang, Baoguo Sun

**Affiliations:** 1 Beijing Key Laboratory of Flavor Chemistry, Beijing Technology and Business University, Beijing 100048, China; qhysdeqj@126.com (Z.W.); taotaodeleyuan@126.com (T.N.); wwwenqian1998@163.com (W.W.); chenht@th.btbu.edu.cn (H.C.); wsq930312@163.com (S.W.); sunbg@btbu.edu.cn (B.S.); 2 China Food Flavor and Nutrition Health Innovation Center, Beijing Technology and Business University, Beijing 100048, China

**Keywords:** pork tenderloin, stir frying, quality attributes, volatile compounds, correlation

## Abstract

Volatile compounds and physicochemical properties of meat are significantly changed by cooking processes. This study explored the influence of different stir-frying temperatures and times on the dynamic changes of the physicochemical characteristics and volatiles of pork tenderloin and determined the correlation between them. Results showed that time played more of a role than temperature. At the same temperature, the water content decreased (*p* < 0.05) and the cooking loss increased (*p* < 0.05) with stir-frying time extending. The L* value and the b* value showed first an increasing and then decreasing trend (*p* < 0.05), while the a* value significantly increased (*p* < 0.05). The higher the cooking temperature of sample, the faster the indexes changed. In stir-fried samples, 50 volatiles were identified. Correlation analysis showed that among the quality attributes, b* value and water content had the strongest impact on volatiles. The water content was negatively correlated with most of the compounds attributed to the desired aroma of stir-fried samples, while the correlation between the b* value and these volatiles was positive. Hence, changes in the types and contents of volatiles in stir-fried pork tenderloin could be predicted by detection of b* value and water content.

## 1. Introduction

Over the last ten years, the need for the industrialization of Chinese cuisine has been growing steadily [1]. Pork is one of the most consumed meats worldwide [2]. Pork tenderloin, due to its tenderness and taste, has always been the basic ingredient of traditional stir-frying dishes, such as fish-flavored pork, in China. Aroma is one of the most crucial quality attributes which greatly affects consumer acceptance and preference [3]. The monitoring of aroma is helpful in determining the end point of the cooking process and optimizing the cooking parameters. It is of significance for improvement in the quality of pork tenderloin dishes and the development of intelligent manufacturing equipment.

The aroma qualities of meat are mainly attributed to volatile substances [4]. Aroma quality assessment based on volatile analysis methods such as gas chromatography-mass spectrometry (GC-MS) has been widely used. Wang et al. [5] investigated the influence of different heating modes on the volatiles and aroma of pork belly and found that balanced distribution and rich contents of volatiles contributed to a better aroma quality. Yang et al. [6] explored the effect of different cooking methods on the aroma compounds in pork loin. The results showed that aldehyde and pyrazine were the key contributors of cooked pork aroma. Chang et al. [7] analyzed the key aroma components in pork broth stewed for different times. Finally, the aroma profile under the optimum stewing time was obtained. Wang et al. [8] identified 12 aroma-active compounds in pork broth by gas chromatography–olfactometry–mass spectrometry and found the broth cooked at 96 °C possessed a strong meaty aroma. However, the analysis of volatile substances under different cooking processes is cumbersome, which is inapplicable in online aroma monitoring of automatic cooking in industrial production.

The changes in volatile compounds are normally accompanied by variations of other quality attributes such as color and water content in meat processing. Wang et al. [9] investigated the changes of texture, color, and volatiles of steamed pork loins. The results showed that hardness, a*, and b* showed an increasing trend with cooking time extending, whereas the L* value and the content of volatiles first increased and then decreased. Zhao et al. [10] examined the dynamic changes in the volatile chemicals and physicochemical properties (gel strength, whiteness, and hardness) of tilapia sausage in the fermentation process and found that the main physical indexes and typical volatile compounds were both significantly enhanced after fermentation. Lee et al. [11] showed that the effect of mild heat treatment of beef tenderloin reduced the formation of meat aroma but caused a remarkable increase in appearance and texture. Compared with the determination of volatiles, the analysis of quality indicators is relatively convenient. Obtaining the correlation data between them could be useful for realizing aroma prediction and quality control in the industrialized production of Chinese cuisine. Related studies have not been reported yet.

Therefore, the aims of this study were to (1) investigate the main physicochemical features that directly impact cooked pork tenderloin quality, such as the water content, cooking loss, color, and volatile compounds and (2) analyze the correlation between volatile compounds and other quality attributes in pork tenderloin in response to different stir-frying processes, which would provide data support for aroma prediction based on physicochemical indexes.

## 2. Materials and Methods

### 2.1. Materials and Chemicals

The fresh pork tenderloin, “Luhua” soybean oil, and food-grade sodium chloride were all purchased from Wu Mart supermarket in Beijing, China. The pork tenderloin was obtained from Delisi Food Co., Ltd. (Beijing, China), freshly cut and packaged in a modified-atmosphere box. To avoid the impact of storage, the samples were purchased on the same day the experiment was conducted and stored at 4 °C for a maximum of 4 h. The n-alkanes (C6–C30) used for retention indices calculation and the internal standard chemical, 2-methyl-3-heptanone, were provided by Sigma-Aldrich (Shanghai, China).

### 2.2. Sample Preparation

The pork tenderloin was washed, cleaned, and then cut into long strips of about 4.00 × 1.00 × 1.00 cm; the average weight was 3.50 ± 0.50 g.

To stir fry the tenderloin, fresh pork samples (20.00 ± 1.00 g) were put into a frying pan with soybean oil (5.00 ± 0.50 g). In this study, pork tenderloin was fried at temperatures of 140 °C, 160 °C, 180 °C, 200 °C, and 220 °C. The frying times at each temperature were 50 s, 100 s, 150 s, 200 s, and 250 s. A total of 25 groups of pork tenderloin samples with different time and temperature conditions were obtained. The cooking treatment was repeated three times. Subsequently, the physicochemical qualities, sensory scores, and volatiles of the samples were obtained. Raw meat of the same size was served as the control group.

### 2.3. Cooking Loss and Water Content

After stir frying, the temperature of samples was decreased to 25 °C. Then, the samples were chopped to measure cooking loss value and water content. The weight difference between the samples before and after stir frying was used to calculate cooking loss [12]. Water content was measured by direct drying method according to the PRC National Standard 5009.3-2016 [13].

### 2.4. Color

The surface color of samples was measured with a CR-1000 Plus chromaticity meter (Konica Minolta, Tokyo, Japan). Fresh-cut pork slices were taken as the control group. Each group contained at least three slices. For each slice, at least three different positions were measured. The color parameters were determined by averaging the values of the three test samples [14]. Color of the pork tenderloin sample was expressed as the values of lightness (L*), redness (a*), and yellowness (b*).

### 2.5. Sensory Evaluation

Twelve panelists (five males and seven females, 20–24 years old) were recruited from Beijing Key Laboratory of Flavor Chemistry of Beijing Technology and Business University. All experimental protocols were approved by the Ethics Committee of Beijing Business and Technology University, and informed consent was obtained from all subjects involved in the study. A sensory evaluation standard for stir-fried pork tenderloin was developed based on the panelists’ description and discussion of the samples’ sensory characteristics before the evaluation [15]. Four attributes were evaluated in the sensory part: color (golden, yellow or light brown, white or dark brown), texture (moderately tender, medium tough or tender, extremely tough or tender), aroma (rich aroma, common aroma, or light aroma) and overall flavor (good flavor, general flavor, or poor flavor). The selected attributes were scored by the panelists. The score scale ranged from 1 to 9. The scoring criteria is shown in Table S1. In this study, the stir-fried samples were placed in a thermostatic container at 60 °C. All panelists completed sensory evaluation within 30 min. To prevent the sequence effect, three random numbers were used to encrypt each sample [16]. The whole evaluation process was conducted in a specialized sensory evaluation laboratory. The team members did not contact and communicate with each other, and they rinsed their mouths with clear water between different samples [17].

### 2.6. Volatile Compounds Analysis

Volatile compounds were extracted by solid-phase micro-extraction (SPME) methods. Each of the 6.00 g stir-fried pork tenderloin samples was cut into about 2 mm granules, 10 μL internal standard (2-methyl-3-heptanone, 16.32 mg/μL) was added to them, and they were placed into a 40 mL headspace vial. Then, the headspace vial was sealed with a polytetrafluoroethylene/silicone septum and equilibrated in a water bath at 45 °C for 20 min to reach the equilibrium state. A SPME fiber coated with divinylbenzene/carboxen/polydimethylsiloxane (DVB/CAR/PDMS, 50/30 µm, Supelco, Bellefonte, PA, USA) was used to adsorb volatiles for 30 min. Eventually, volatiles were thermally desorbed at GC-MS injection port at 250 °C for 5 min.

The volatile compounds were analyzed by Thermo Fisher Trace 1310 gas chromatography (Thermo Fisher Scientific, Waltham, MA, USA) combined with a mass selection detector (Thermo Fisher Scientific, Waltham, MA, USA). DB-WAX column (30 m × 0.25 mm × 0.25 μm; Thermo Fisher Scientific, Waltham, MA, USA) was used to separate different volatiles. Helium (purity of 99.99%) was used as the carrier gas, and the flow rate was 1.0 mL/min. The programmed temperature conditions were as follows: the initial temperature of oven was 40 °C kept for 2 min, and then the oven temperature was ramped to 230 °C at 5 °C/min and maintained for 4 min. The mass spectrometry detector conditions were as follows. The ionization energy was 70 eV. The ion source and transfer line temperatures were set as 250 °C and 240 °C, respectively. The mass scanning ranges were 45–450 *m*/*z*.

Volatile compounds were tentatively identified by comparison with the mass spectrum of existing data in NIST 14.0 databases. Retention indexes (RIs) methodologies were also applied using n-alkane (C6–C30) as standards under the same GC conditions. Compounds with a matching probability higher than 80% and an RI difference value less than 20 were considered [18]. The concentration of each volatile compound was determined by comparing the peak area of the target component with that of the internal standard [19]. The average of the three replicates represented the final results.

### 2.7. Statistical Analysis

All of the measurements were repeated in triplicate. One-way analysis of variance (ANOVA) with a significance level of *p* < 0.05 was performed using SPSS Statistics 19.0 (IBM Corp., Chicago, IL, USA). Tukey’s test was used to assess significant differences (*p* < 0.05) between separate groups. Shapiro–Wilk test was applied for testing normality, and Levene’s test (*p* < 0.05) was used to test variance homogeneity. When the variance did not meet the hypothesis of homogeneity, Welch’s test was applied for post hoc pairwise comparisons. Partial least squares regression analysis between physicochemical characteristics and volatile compounds was analyzed using XLSTAT 2018 (Addinsoft, New York, NY, USA). The correlation heatmap was carried out by using tools in Hiplot Pro 2.0 (Shanghai Tengyun Biotechnology Co., Ltd., Shanghai, China). Other graphics were drawn by Origin 2021 (OriginLab Corp., Northampton, MA, USA).

## 3. Result and Discussion

### 3.1. The Changes of Quality Attributes in Stir-Fried Pork Tenderloin

#### 3.1.1. Cooking Loss and Water Content

**Figure 1:**
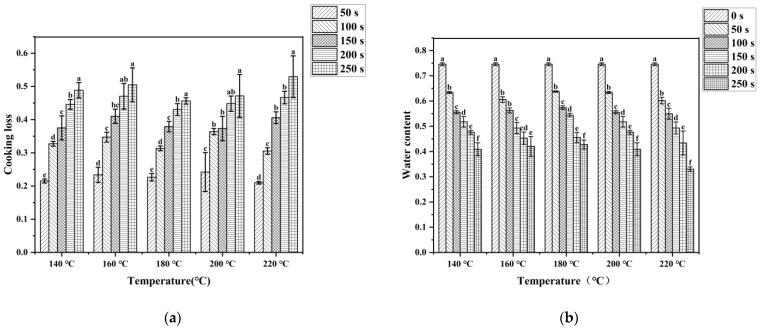
Cooking loss (**a**) and water content (**b**) of pork tenderloin under various cooking conditions. Values bearing different lowercase letters within the same temperature were significantly different (*p* < 0.05).

The water content and cooking loss of stir-fried pork tenderloin are shown in Figure 1. It can be observed that the longer stir-frying time resulted in a higher cooking loss and a lower water content. This was consistent with previous research showing that cooking loss in rabbit meat increased with longer heating times [20]. The water content of the raw pork tenderloin was the highest, about 75%, and decreased to 33% at most. The higher the cooking temperature of sample, the faster the water content decreased. Due to heat processing, protein denaturation and contraction reduced the water-holding capacity of muscle fibers, resulting in a gradual loss of water with the increase in stir-frying time. The moisture content in pork has an important influence on the texture, appearance, and flavor of pork meat. Meat product with slightly higher moisture content has a better chewiness and juiciness, which were considered to be important factors in determining the quality of meat [21]. A previous study showed that during steak frying, the sensory score of juiciness would significantly decrease with the increase in moisture loss. When the moisture content dropped too low, it would lead to a decrease in palatability [22]. Considering the results, only when the water content of cooked pork is not lower than 40% could a relatively better edible quality be found. It was shown in Figure 1b that when the stir-frying time reached 250 s, the water content of most samples decreased to about 40%, so the stir frying time should not exceed 250 s.

#### 3.1.2. Color

**Figure 2:**
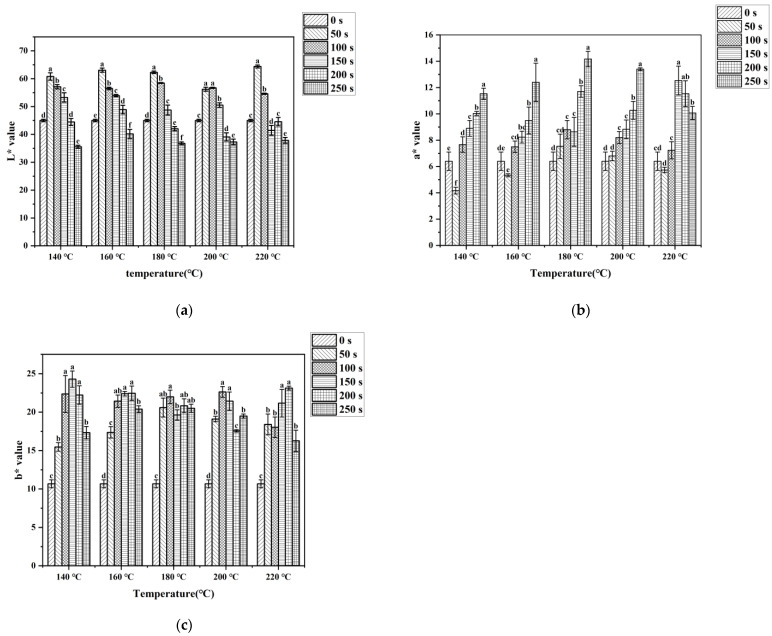
L* value (**a**), a* value (**b**), and b* value (**c**) of pork tenderloin under various cooking conditions. Values bearing different lowercase letters within the same temperature were significantly different (*p* < 0.05).

Cooking processes in both industry and the household have an influence on meat color, which directly influences purchasing desire [23,24]. The color change in meat products during cooking mainly depends on the thermal denaturation of myoglobin. Globin denaturation in myoglobin leads to the formation of denatured globin hemichrome, which is responsible for the dull-brown color in cooked meats. In addition, Maillard reaction products formed on the meat surface also contributed to the color variation [25,26]. L* value is one of the most vital indicators of evaluating meat product quality. The average L* value of uncooked pork tenderloin was 45.00, as indicated in Figure 2a. Compared to the control group, the L* value increased significantly within 50 s in the stir-frying process. After that, with the extension of stir-frying time, the L* value began to decline gradually. When the L* value decreased to 40, the surface color of pork tenderloin turned almost black. The mean a* and b* values of the control group were, respectively, 6.4 and 10.67. The a* value of the samples exhibited a remarkable increasing trend with time, while showed a decreasing trend from 200 s to 250 s at 220 °C. The change trend of the L* value and the a* value was in agreement with that reported for fried shrimp at different temperatures [27]. The b* value exhibited a trend of initially rising and then dropping, and the b* value of each treatment was higher than that of the control group. During the stir-frying process, due to the denaturation of myoglobin and the Maillard reaction, the pork tenderloin changed from white to golden yellow and, finally, to dark brown. Under the same cooking conditions, all b* values were greater than a* values, which showed that the yellow color of stir-fried samples occupied a dominant position. The data in Figure 2 showed that when the stir-frying time reached 250 s, the L* value of the pork tenderloin surface would drop below 40. In high-temperature, short-time cooking, time played more of a role than temperature in the color change in cooked meat. Therefore, too much stir-frying time is not recommended.

### 3.2. Sensory Evaluation

The four attributes (color, tenderness, aroma, and overall flavor) of each pork tenderloin sample were evaluated by a team of 12 well-trained individuals. The sensory qualities of samples were shown to change with different temperatures and stir-frying times (Table 1). As shown in Table 1, the color score of pork tenderloin samples presented an earlier increasing and later decreasing trend (*p* < 0.05) and became relatively higher from 150 to 200 s. During the stir-frying process, with the denaturation of myoglobin and the Maillard reaction with the loss of water, the meat color changed from white to golden yellow, then turned to dark brown. Tenderness has been determined to be one of the vital factors affecting acceptability for consumers [28]. In terms of the tenderness of the samples, they showed a decreased trend with the increase in stir-frying time. With regard to odor, the scores of stir-fried pork tenderloin showed a remarkable increasing trend. With the prolongation of cooking time, the Maillard reaction occurs, and lipid oxidation produces more flavor substances, which makes the aroma more pleasant [29]. For the overall flavor, the scores elevated gradually with increasing stir-frying time. However, deep stir-fried samples showed a decreasing trend in overall flavor score. In conclusion, when the stir-frying time was 150–200 s, the cooked pork tenderloin had the golden color, tender texture, and pleasant flavor of stir-fried dishes.

### 3.3. Volatile Compounds

Odor is a crucial sensory attribute for meat products since it influences consumers’ initial perception and acceptance of the product. The relationship between volatile components and odor characteristics has been well documented. To identify the difference of odor in pork tenderloin during stir frying, the SPME-GC-MS method was used to analyze the volatiles in samples stir fried at 140 °C and 220 °C for 100 s, 150 s, 200 s, and 250 s. As shown in Table 2, 51 volatile compounds in pork tenderloin were tentatively identified, including fifteen aldehydes, thirteen alcohols, five ketones, four esters, one terpenoid, two furans, three phenols, five pyrazines, one ether, and two sulfur-containing compounds. There were 24 volatiles identified in raw pork and 50 volatiles identified in stir-fried samples, which indicated the notable effect of stir frying on the generation of volatile compounds.

Aldehydes are primarily produced by the oxidation of lipids and are characterized by pleasant odors like malt, grass, cheese, and fruit [30]. It has been demonstrated that these compounds significantly improved the flavor of pork [31]. In this investigation, aldehydes were the most prevalent class of volatile chemicals found in stir-fried pork tenderloin. Among them, relatively high contents of aliphatic aldehydes, including hexanal, heptanal, octanal, and nonanal with grass and fat odors, were found. In general, over half of the volatiles in cooked meat are formed by lipid oxidation and degradation. Different cooking conditions lead to different lipid oxidation pathways, influencing the volatiles of meat products. Oleic acid is a common class of monounsaturated fatty acids found in meat, whereas arachidonic acid and linoleic acid are common polyunsaturated fatty acids. Compounds including octanal, nonanal, and decanal are produced by oleic acid oxidation. Hexanal and (*E*)-2-nonanol are oxidized from linoleic acid [32]. In fresh pork tenderloin samples, aldehydes were undetected or detected with a very low content. Under less intense cooking conditions, aldehydes in cooked pork tenderloins were present in the highest proportion. However, the relative content decreased as time went on, especially in samples cooked at a lower temperature. The changes (increase followed by a decrease) in the levels of aldehydes could be attributed to lipid oxidation and heat-induced evaporation.

Alcohol formation is also attributed to polyunsaturated fatty acid oxidization and degradation [33]. Among the detected 13 alcohol compounds, 2-ethyl-1-hexanol, 1-octen-3-ol, pentanol, hexanol, heptanol, and 1-octanol were the main alcohol compounds. These thirteen volatiles might be grouped into two clusters, as Table 1 illustrates, according to the pattern of fluctuations in their concentration in the samples. Volatiles in the first group, including 2-ethyl-1-hexanol, linalool, octanol, 2,3-butanediol, hexanol, nonanol, and dodecanol, were found in a high content in the control sample. However, the content of these compounds decreased in the fried samples. One possible explanation for the elimination of these volatiles might be evaporation during high-temperature cooking. On the other hand, volatile substances in the second cluster, including 1-octen-3-ol, pentanol, heptanol, (*E*)-2-octen-1-ol, and 4-isopropoxybutanol had a higher content in stir-fried samples compared to those in raw meat, which could be attributed to lipid oxidation during the cooking process. 1-Octen-3-ol is an odorant that is typically found in meat products. Its odor is similar to that of mushroom. Throughout the cooking process, the concentration of 1-octen-3-ol remained high in our investigation. Because of its large quantity and relatively low threshold value, 1-octen-3-ol might be regarded as a key distinctive flavor component in cooked meat [34].

Ketones are usually generated by the oxidation of unsaturated fatty acids or through the degradation of amino acids, and they have a distinctive fruity and floral aroma [35]. A total of 5 ketones were discovered throughout the cooking stage, including acetoin (cheese aroma), 6-methyl-5-hepten-2-one (fruit and grass aroma), 2-undecanone (fruit and fatty aroma), 6,10-dimethyl-5,9-undecadien-2-one (spicy aroma), and 1-(2,4-dimethylphenyl)-ethanone (floral aroma), which might contribute to the aroma formation of stir-fried pork tenderloin because of their lower thresholds. Among ketones, except for acetoin, which was more abundant in raw pork, the content of the others increased and then decreased after cooking. This might be because ketones could serve as substrates to react with amino acids, peptides, proteins, and other components to form heterocyclic chemicals, which are indirectly attributed to the development of pork flavor [36,37].

Esters are formed through the esterification of organic acids and alcohols. Only four esters with relatively low contents were found in this investigation. γ-Nonanolactone was found in raw pork samples, whereas the other three esters were generated throughout the successive stir-frying phases.

Furan derivatives are characteristic compounds generated in the Maillard reaction and fat degradation processes [27]. In all samples, only two furans were identified. 2(5*H*)-Furanone is a Maillard reaction product with a typical caramel-like and roasted aroma. 2-Pentylfuran, which has a beany and fruity smell, contributed significantly to the overall flavor of pork and might be caused by the oxidation of linoleic acid [8,38]. Throughout the stir-frying stage of pork tenderloin, the content of 2-pentyl-furan in samples was significantly increased after cooking.

The heterocyclic compounds containing sulfur are the most significant substances created by the Maillard reaction and contribute to the meaty, savory, and roasted flavor characteristics of cooked meat [29]. Sulfur-containing compounds have a low threshold and a vital influence on the flavor formation of pork at low content. In the study, one sulfur-containing heterocyclic compound, namely, 4-methyl-5-thiazoleethanol, could be detected with the prolongation of cooking time. The reason why the other sulfur compounds were not detected might be that the content of sulfur amino acids in pork tenderloin is too low, which leads to a low content of sulfur-containing compounds generated during the reaction. It was also possible that the method applied in this investigation was insufficient to detect these sulfur components [39].

Pyrazines have a distinctive roasted smell. They are typical Maillard reaction products which are formed by the aldehyde condensation reaction and Strecker degradation during cooking [27,40]. Pyrazines were not identified in the control group. During stir frying, the content of pyrazines could judge the progress of the Maillard reaction [31]. The findings demonstrated that heating at a higher temperature and, to a lesser extent, prolonged cooking time, promoted the formation of volatile compounds from the Maillard reaction, which stimulated desirable flavors.

The most likely source of phenolic compounds in pork tenderloin is animal feed [41]. The phenolic compounds were found in raw pork samples and decreased after cooking.

### 3.4. The Correlations between Physical Index and Volatile Compounds

**Figure 3:**
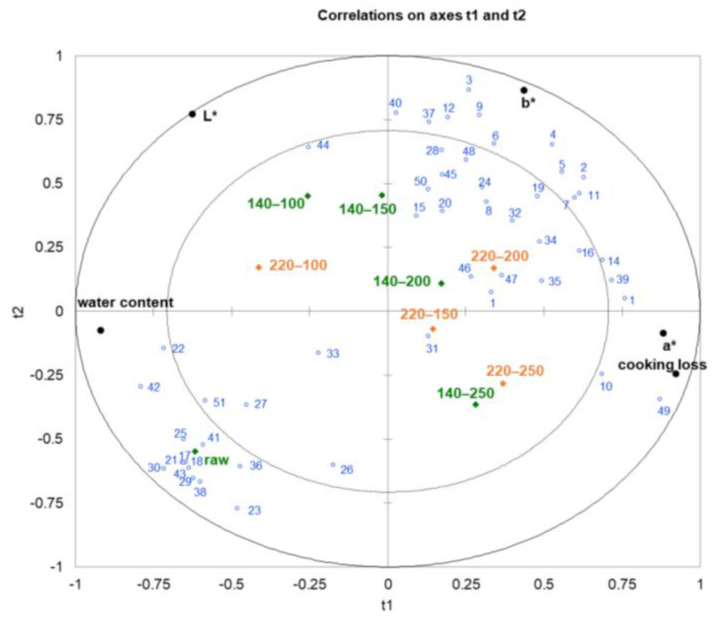
Partial least squares regression (PLSR) analysis between physical indexes and volatile compounds in pork tenderloin stir fried at 140 °C (indicated by green) and 220 °C (indicated by orange). The No. (indicated by blue) in the figure is in accordance with the No. of compounds in Table 2.

Meat’s physicochemical characteristics and volatile components can be drastically altered by heat treatment. The aroma attributes’ changes are accompanied by variations of other quality attributes. Normally, the detection of volatile substances in meat processing is cumbersome, and the determination of quality indicators is relatively convenient. Obtaining the correlation data between them could better realize the aroma prediction in the industrial production process. In this study, the correlations among quality attributes and volatile components were studied using partial least squares regression (PLSR) analysis, and the results are shown in Figure 3. As shown in Figure 3, the physical index and most volatile compounds were located between the inner and outer ellipses, demonstrating that they were remarkably explained by the PLSR model [42]. The x variables (R^2^ X = 0.894) explained the variation in the y variables (R^2^ Y = 0.505) according to the first two factors in the correlation loading plot for the volatile chemicals and the physicochemical indices. Factor 1 corresponded to increasing sample stir-frying times. Factor 1 explained a majority of the variance in both data sets, indicating that the effects of time in stir-frying processes were responsible for the majority of the covariance between the two data sets.

Cooking loss, a*, and b* descriptors were positively correlated with factor 1 and were associated with samples stir fried ≥150 s under 220 °C and samples stir fried ≥200 s under 140 °C, while water content and L* value were associated with the control and stir-fried <150 s samples and negatively correlated with factor 1 (Figure 3). Regarding factor 1, volatile chemicals in the headspace were divided into two primary groups that correlated with early and late stir-frying periods. The majority of volatile substances negatively correlated with factor 1 and positively correlated with water content attribute were mainly alcohols and phenols. These volatiles showed high correlation with raw samples. The other groups of volatiles most positively associated with factor 1, cooking loss, a*, and b* values mainly included volatiles particularly those formed from Maillard reactions and lipid oxidation, such as pyrazines, sulfur-containing components, aldehydes, five to eight-carbon alcohols, six to eight-carbon esters, and furans. As shown in Figure 3, the volatiles in the second group were plotted in the vicinity of samples 140–200, 220–150, and 220–200, indicating these volatiles and samples were correlated with each other.

**Figure 4:**
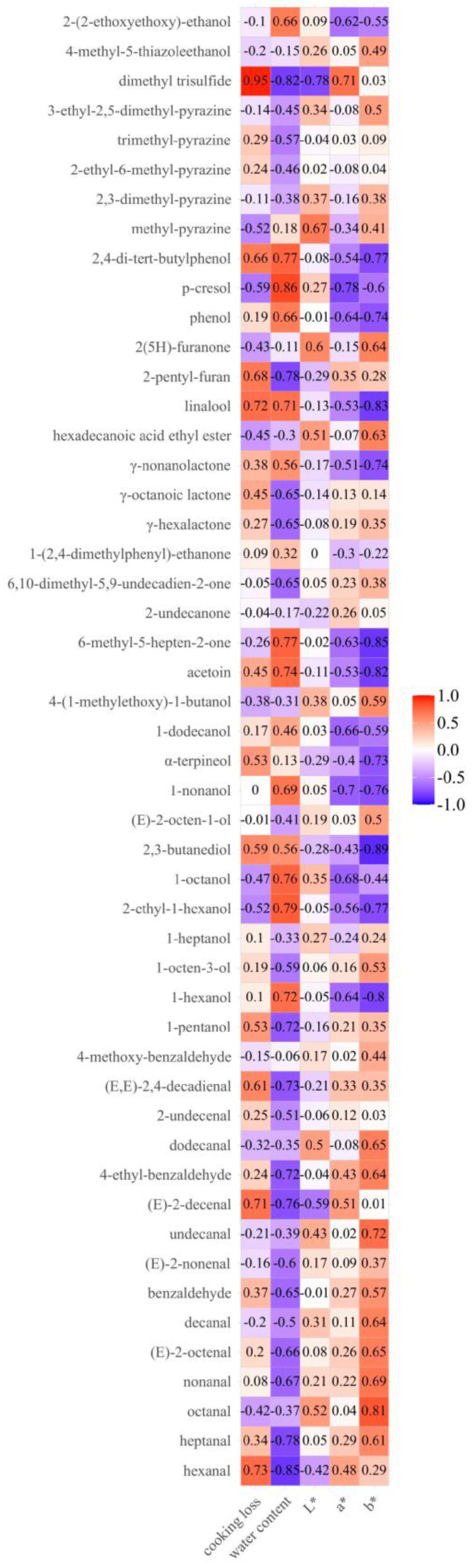
The correlation heatmap between physical index and volatile compound.

The correlation coefficients between the 50 detected volatiles and quality attributes, including L*, a*, b*, water content, and cooking loss, are shown in Figure 4. Among the quality attributes, b* value and water content had the strongest impact on the volatiles, while cooking loss, L* value, and a* value only showed strong correlations with a few volatile compounds, including hexanal, (*E*)-2-decenal, 1-nonanol, linalool, p-cresol, and dimethyl trisulfide. As shown in Figure 4, aldehydes showed a negative correlation with water content, while being positively correlated with the b* value. During the stir-frying process, the heat transferred rapidly to the pork samples. With an increased temperature, the water decreased, and the surface color changed. This process accelerated the oxidation reaction of fatty acids and resulted in the accumulation of aldehydes. The correlation coefficients for indexes of water content and b* value with alcohols were also higher than the others. Among alcohols, 1-pentanol, 1-octen-3-ol, 1-heptanol, (*E*)-2-octen-1-ol, and 4-(1-methylethoy)-1-butanol were negatively related to water content and showed a positive correlation with the b* value. As the products of lipid oxidation and heat degradation, these alcohols accumulated and helped to form the desired aroma of stir-fried samples. Among the ketones with high correlation with the physicochemical indexes, acetoin and 6-methyl-5-hepten-2-one were positively correlated with water content and negatively related to b* value. This could be linked to the abundance of these compounds in raw pork samples. The compound of 6,10-dimethyl-5,9-undecadien-2-one was negatively correlated to water content, but there was no obvious correlation between 6,10-dimethyl-5,9-undecadien-2-one and b* value. For esters, γ-nonanolactone was positively correlated with water content and had a negative correlation with b* value. γ-Nonanolactone, with milk aroma, was usually detected in the raw pork. However, other esters presented negative correlations with water content, which might be due to their formation through the esterification of organic acids and alcohols throughout the successive stir-frying phases. Phenolic compounds also showed positive correlations with water content and negatively correlated with b* value. The most likely source of phenolic compounds in pork tenderloin is animal feed, and they were found in raw pork samples and decreased after cooking. Pyrazines were negatively related to water content, but the correlation between pyrazines and physicochemical attributes was relatively small. This might be due to the coexistence of multiple reactions in the food processing system and the complexity and interaction between different reactions, so the compounds derived from the same reaction type or the same reaction pathway cannot present an ideal correlation. To sum up, the water content and b* value could be used as potential indicators of flavor in pork tenderloin during stir frying.

## 4. Conclusions

This paper showed the influence of different stir-frying temperatures and times on the dynamic changes of the physicochemical characteristics and volatile compounds of pork tenderloin and determined the correlation between them. The main physical indexes (water content, cooking loss, brightness, yellowness, and redness) and characteristic volatiles of pork tenderloin changed significantly with different stir-frying temperatures and times. Volatiles, particularly those formed from Maillard reactions and lipid oxidation, such as aldehydes, five to eight-carbon alcohols, six to eight-carbon esters, and pyrazines, accumulated and helped to form the desired aroma of the stir-fried samples. Correlation analysis showed that among the quality attributes, b* value and water content had the strongest impact on these volatiles. The water content was negatively correlated with most of the compounds in stir-fried samples, while the correlation between the b* value and these volatiles was positive. The study provides data support for volatile prediction based on physicochemical indexes. This is an important basis for future attempts to realize flavor prediction and quality control in the industrial production of Chinese dishes and to provide ideas for developing an online flavor control system.

## Figures and Tables

**Table 1:** Sensory evaluation of stir-fried pork tenderloin under different cooking processes.

Cooking Conditions		Sensory Scores ^a^			
Temperature (°C)	Time (s)	Color	Tenderness	Aroma	Overall Flavor
140	50	4.00 ^b^	7.00 ^ab^	4.25 ^c^	5.13 ^a^
	100	4.38 ^b^	7.50 ^a^	5.38 ^abc^	6.38 ^a^
	150	5.00 ^b^	6.38 ^abc^	5.25 ^bc^	6.25 ^a^
	200	6.88 ^a^	6.13 ^bc^	6.63 ^a^	6.63 ^a^
	250	6.25 ^a^	5.38 ^c^	6.50 ^ab^	6.50 ^a^
160	50	3.50 ^b^	6.63 ^a^	5.25 ^a^	5.38 ^ab^
	100	6.00 ^a^	6.88 ^a^	5.25 ^a^	5.38 ^ab^
	150	6.63 ^a^	5.25 ^b^	4.50 ^a^	6.75 ^a^
	200	3.38 ^b^	3.13 ^c^	5.75 ^a^	4.75 ^b^
	250	4.25 ^b^	3.50 ^c^	5.63 ^a^	5.50 ^ab^
180	50	3.25 ^c^	6.25 ^ab^	5.50 ^a^	5.38 ^a^
	100	4.00 ^bc^	6.88 ^a^	5.88 ^a^	5.75 ^a^
	150	6.38 ^a^	4.88 ^b^	6.25 ^a^	6.25 ^a^
	200	5.75 ^ab^	5.13 ^b^	6.00 ^a^	6.75 ^a^
	250	5.13 ^abc^	2.75 ^c^	6.75 ^a^	6.25 ^a^
200	50	5.13 ^a^	7.88 ^a^	6.13 ^a^	6.13 ^a^
	100	5.00 ^a^	6.63 ^ab^	5.50 ^a^	5.88 ^a^
	150	6.13 ^a^	5.50 ^bc^	6.63 ^a^	6.38 ^a^
	200	5.13 ^a^	4.25 ^c^	7.00 ^a^	6.63 ^a^
	250	5.00 ^a^	4.63 ^c^	6.13 ^a^	6.50 ^a^
220	50	4.00 ^c^	7.25 ^a^	6.00 ^ab^	6.13 ^a^
	100	4.75 ^bc^	6.75 ^a^	5.75 ^b^	5.88 ^a^
	150	6.38 ^ab^	6.50 ^a^	5.38 ^b^	6.13 ^a^
	200	6.88 ^a^	5.75 ^ab^	6.63 ^ab^	7.00 ^a^
	250	5.88 ^ab^	4.25 ^b^	7.88 ^a^	7.25 ^a^

^abc^ Values bearing different lowercase letters within the same temperature are significantly different (*p* < 0.05).

**Table 2:** Relative concentration (μg/Kg) of volatile compounds in stir-fried pork tenderloin under different cooking processes.

No.	Compounds	Identification Methods ^a^	RI ^b^		Concentration (μg/Kg) ^c^									
			Calculation	Literature	Raw	140 °C 100 s	140 °C 150 s	140 °C 200 s	140 °C 250 s	Raw	220 °C 100 s	220 °C 150 s	220 °C 200 s	220 °C 250 s
	aldehydes													
1	hexanal	MS RI	1067	1078	73.97 ^b^	854.68 ^ab^	790.97 ^ab^	1730.12 ^a^	1362.79 ^a^	73.97 ^b^	828.14 ^ab^	732.98 ^ab^	968.51 ^ab^	1717.63 ^a^
2	heptanal	MS RI	1182	1181	0 ^b^	70.72 ^a^	65.59 ^a^	64.67 ^a^	59.51 ^a^	0 ^c^	45.05 ^ab^	31.17 ^bc^	57.90 ^ab^	77.75 ^a^
3	octanal	MS RI	1286	1286	0 ^c^	128.28 ^a^	115.75 ^ab^	84.10 ^ab^	63.14 ^b^	0 ^c^	64.70 ^ab^	45.63 ^b^	76.42 ^a^	67.37 ^ab^
4	nonanal	MS RI	1390	1390	31.40 ^b^	518.49 ^a^	519.87 ^a^	423.28 ^a^	358.78 ^a^	31.40 ^c^	304.78 ^ab^	257.84 ^b^	369.30 ^ab^	505.43 ^a^
5	(*E*)-2-octenal	MS RI	1424	1434	0 ^b^	15.00 ^a^	18.38 ^a^	21.25 ^a^	13.54 ^a^	0 ^c^	11.47 ^b^	8.84 ^b^	10.94 ^b^	17.27 ^a^
6	decanal	MS RI	1492	1485	0 ^c^	23.53 ^ab^	25.57 ^a^	13.58 ^b^	19.09 ^ab^	0 ^c^	13.35 ^ab^	9.33 ^b^	12.09 ^ab^	17.13 ^a^
7	benzaldehyde	MS RI	1512	1508	6.41 ^b^	32.69 ^a^	32.73 ^a^	29.46 ^ab^	25.50 ^ab^	6.41 ^c^	10.56 ^c^	15.38 ^bc^	22.88 ^ab^	34.06 ^a^
8	(*E*)-2-nonenal	MS RI	1528	1530	0 ^b^	6.71 ^a^	3.66 ^a^	6.83 ^a^	5.11 ^a^	0 ^b^	8.10 ^a^	3.66 ^ab^	4.64 ^a^	7.19 ^a^
9	undecanal	MS RI	1599	1587	0 ^c^	4.66 ^ab^	7.05 ^a^	3.23 ^bc^	2.43 ^bc^	0 ^c^	2.66 ^ab^	1.75 ^b^	3.29 ^a^	3.94 ^a^
10	(*E*)-2-decenal	MS RI	1638	1630	0 ^c^	0 ^c^	0 ^c^	15.38 ^a^	10.83 ^b^	0 ^c^	10.22 ^b^	7.05 ^b^	12.41 ^ab^	18.29 ^a^
11	4-ethyl-benzaldehyde	MS RI	1699	1719	0 ^d^	2.29 ^c^	2.69 ^bc^	3.50 ^a^	3.11 ^ab^	0 ^b^	2.48 ^a^	1.82 ^ab^	2.21 ^a^	2.43 ^a^
12	dodecanal	MS RI	1705	1710	0 ^b^	4.54 ^a^	4.41 ^a^	2.34 ^ab^	2.03 ^ab^	0 ^c^	2.04 ^ab^	1.24 ^bc^	1.85 ^ab^	2.98 ^a^
13	2-undecenal	MS	1746	-	0 ^b^	6.95 ^a^	0 ^b^	0 ^b^	0 ^b^	0 ^d^	5.40 ^bc^	3.49 ^cd^	9.01 ^ab^	13.84 ^a^
14	(*E*,*E*)-2,4-decadienal	MS RI	1802	1813	0 ^d^	16.29 ^c^	39.03 ^a^	21.06 ^b^	16.88 ^bc^	0 ^c^	5.07 ^c^	8.96 ^c^	33.69 ^b^	53.78 ^a^
15	4-methoxy-benzaldehyde	MS RI	2012	2011	0 ^b^	0.55 ^b^	0.75 ^b^	1.91 ^a^	0.51 ^b^	0 ^b^	0.49 ^a^	0.30 ^ab^	0 ^b^	0 ^b^
	alcohols													
16	1-pentanol	MS RI	1252	1252	29.62 ^b^	60.22 ^ab^	85.01 ^a^	88.68 ^a^	68.40 ^ab^	29.62 ^b^	52.42 ^ab^	38.00 ^b^	60.92 ^ab^	102.87 ^a^
17	3-methyl-2-buten-1-ol	MS RI	1325	1320	0.01 a	0 ^a^	0 ^a^	0 ^a^	0 ^a^	0.01 ^a^	0 ^a^	0 ^a^	0 ^a^	0 ^a^
18	1-hexanol	MS RI	1354	1369	47.06 ^a^	14.49 ^b^	18.99 ^b^	20.22 ^b^	16.40 ^b^	47.06 ^a^	18.92 ^b^	12.94 ^b^	12.04 ^b^	20.55 ^b^
19	1-octen-3-ol	MS RI	1449	1451	42.97 ^a^	136.01 ^b^	191.93 ^b^	185.01 ^b^	145.14 ^b^	42.97 ^c^	114.99 ^b^	88.15 ^b^	94.33 ^b^	168.01 ^a^
20	1-heptanol	MS RI	1455	1447	16.16 ^b^	24.57 ^ab^	31.57 ^a^	19.92 ^ab^	15.06 ^b^	16.16 ^ab^	18.67 ^ab^	11.11 ^b^	20.43 ^ab^	32.57 ^a^
21	2-ethyl-1-hexanol	MS RI	1487	1484	2263.71 ^a^	20.14 ^c^	31.29 ^b^	21.99 ^c^	28.27 ^b^	2263.71 ^a^	37.34 ^b^	18.43 ^b^	20.18 ^b^	17.59 ^b^
22	1-octanol	MS RI	1559	1561	55.27 ^a^	48.70 ^a^	0 ^b^	0 ^b^	0 ^b^	55.27 ^b^	0 ^a^	0 ^a^	0 ^a^	0 ^a^
23	2,3-butanediol	MS RI	1574	1570	133.87 ^a^	0 ^b^	1.09 ^b^	4.60 ^b^	15.86 ^b^	133.87 ^a^	8.43 ^b^	21.45 ^b^	0 ^b^	50.35 ^b^
24	(*E*)-2-octen-1-ol	MS RI	1614	1618	7.20 ^b^	25.32 ^a^	29.13 ^a^	24.15 ^ab^	22.20 ^ab^	7.20 ^c^	13.76 ^b^	12.32 ^bc^	10.21 ^bc^	22.18 ^a^
25	1-nonanol	MS RI	1659	1673	7.53 ^a^	3.07 ^b^	2.85 ^b^	2.19 ^b^	1.95 ^b^	7.53 ^a^	2.15 ^b^	1.35 ^b^	1.41 ^b^	3.51 ^ab^
26	α-terpineol	MS	1694	-	18.50 ^a^	5.08 ^b^	7.59 ^b^	6.67 ^b^	5.47 ^b^	18.50 ^a^	5.24 ^a^	3.15 ^a^	3.95 ^a^	20.09 ^a^
27	1-dodecanol	MS RI	1965	1953	7.10 ^a^	4.04 ^a^	5.21 ^a^	4.50 ^a^	3.91 ^a^	7.10 ^a^	3.35 ^ab^	1.93 ^b^	2.52 ^b^	5.18 ^ab^
28	4-(1-methylethoxy)-1-butanol	MS	2484	-	0 ^c^	0.75 ^bc^	2.10 ^a^	0.87 ^b^	0.99 ^b^	0 ^c^	1.37 ^a^	0.71 ^b^	0.70 ^b^	0.82 ^b^
	ketones													
29	acetoin	MS RI	1279	1273	1486.72 ^a^	1.60 ^b^	5.21 ^b^	23.31 ^b^	47.57 ^b^	1486.72 ^a^	71.56 ^b^	84.53 ^b^	6.24 ^b^	184.13 ^b^
30	6-methyl-5-hepten-2-one	MS RI	1334	1341	19.53 ^a^	4.65 ^b^	2.21 ^b^	2.25 ^b^	3.24 ^b^	19.53 ^a^	5.09 ^b^	3.70 ^b^	1.49 ^b^	5.10 ^b^
31	2-undecanone	MS RI	1594	1599	0 ^b^	0 ^b^	0 ^b^	3.42 ^a^	2.72 ^a^	0 ^b^	2.62 ^a^	1.84 ^a^	0 ^b^	0 ^b^
32	6,10-dimethyl-5,9-undecadien-2-one	MS	1849	-	0 ^b^	1.13 ^a^	0.84 ^a^	0.71 ^a^	1.01 ^a^	0 ^c^	0.86 ^a^	0.79 ^ab^	0.39 ^bc^	1.15 ^a^
33	1-(2,4-dimethylphenyl)-ethanone	MS	2223	-	2.41 ^a^	0 ^b^	2.98 ^a^	1.55 ^ab^	1.26 ^ab^	2.41 ^a^	1.16 ^ab^	0.66 ^b^	0.91 ^b^	1.20 ^ab^
	esters													
34	γ-hexalactone	MS RI	1694	1678	0 ^c^	0.85 ^bc^	2.96 ^a^	2.28 ^ab^	2.06 ^ab^	0 ^d^	1.97 ^ab^	0.89 ^cd^	1.03 ^bc^	2.72 ^a^
35	γ-octanoic lactone	MS RI	1905	1898	0 ^d^	2.35 ^a^	1.96 ^a^	1.31 ^b^	0.78 ^c^	0 ^a^	0.57 ^a^	0.77 ^a^	1.58 ^a^	5.18 ^a^
36	γ-nonanolactone	MS RI	2018	2007	3.70 ^a^	1.06 ^b^	2.11 ^ab^	1.45 ^b^	1.89 ^ab^	3.70 ^a^	1.22 ^b^	0.92 ^b^	1.00 ^b^	1.87 ^ab^
37	hexadecanoic acid ethyl ester	MS RI	2239	2220	0 ^b^	3.67 ^a^	2.63 ^a^	1.96 ^ab^	1.68 ^ab^	0 ^c^	1.58 ^ab^	1.22 ^b^	1.07 ^b^	1.86 ^a^
	terpenoid													
38	linalool	MS RI	1544	1540	23.70 ^a^	1.10 ^b^	0.96 ^b^	1.76 ^b^	2.60 ^b^	23.70 ^a^	1.70 ^b^	1.22 ^b^	1.45 ^b^	3.93 ^b^
	furans													
39	2-pentyl-furan	MS RI	1230	1230	0 ^c^	22.89 ^b^	43.84 ^a^	30.12 ^ab^	24.64 ^b^	0 ^c^	8.26 ^c^	11.91 ^c^	46.90 ^b^	78.27 ^a^
40	2(5*H*)-furanone	MS RI	1741	1767	0 ^c^	1.33 ^a^	1.09 ^ab^	0.45 ^abc^	0.30 ^bc^	0 ^c^	0.26 ^b^	0.32 ^b^	0.34 ^b^	0.48 ^a^
	phenols													
41	phenol	MS RI	1995	1953	7.38 ^a^	2.23 ^b^	3.26 ^b^	2.18 ^b^	2.02 ^b^	7.38 ^a^	1.98 ^b^	1.22 ^b^	1.63 ^b^	3.37 ^ab^
42	p-cresol	MS RI	2073	2078	2.07 ^a^	0.93 ^ab^	1.04 ^ab^	0.62 ^b^	0.51 ^b^	2.07 ^a^	0.70 ^b^	0.41 ^b^	0.41 ^b^	0.58 ^b^
43	2,4-di-tert-butylphenol	MS RI	2305	2312	18.73 ^a^	0 ^b^	0.42 ^b^	0.82 ^b^	0.70 ^b^	18.73 ^a^	0.30 ^b^	0.33 ^b^	0.24 ^b^	0.63 ^b^
	pyrazines													
44	methyl-pyrazine	MS RI	1267	1265	0 ^b^	3.15 ^a^	1.10 ^a^	0 ^b^	0 ^b^	0 ^a^	0 ^a^	0 ^a^	0.22 ^a^	0 ^a^
45	2,3-dimethyl-pyrazine	MS RI	1343	1357	0 ^d^	2.62 ^a^	2.13 ^b^	0.72 ^c^	0.75 ^c^	0 ^b^	0.87 ^b^	0.36 ^b^	0.80 ^b^	2.40 ^a^
46	2-ethyl-6-methyl-pyrazine	MS RI	1383	1382	0 ^d^	3.81 ^a^	2.07 ^b^	0 ^d^	1.02 ^c^	0 ^b^	0.35 ^b^	0 ^b^	0.85 ^b^	5.63 ^a^
47	trimethyl-pyrazine	MS RI	1402	1406	0 ^c^	12.50 ^a^	12.33 ^a^	0 ^c^	7.02 ^b^	0 ^c^	4.01 ^bc^	2.46 ^bc^	5.74 ^b^	23.04 ^a^
48	3-ethyl-2,5-dimethyl-pyrazine	MS RI	1443	1430	0 ^c^	8.90 ^a^	13.76 ^b^	7.08 ^b^	6.94 ^b^	0 ^c^	7.49 ^ab^	2.15 ^bc^	4.26 ^abc^	9.97 ^a^
	sulfur-containing compounds													
49	dimethyl trisulfide	MS RI	1373	1378	0 ^b^	0 ^b^	0 ^b^	0.18 ^a^	0.18 ^a^	0 ^c^	0 ^c^	0.12 ^bc^	0.22 ^ab^	0.30 ^a^
50	4-methyl-5-thiazoleethanol	MS	2299	-	0 ^c^	0 ^c^	1.09 ^a^	0.39 ^b^	0.32 ^b^	0 ^b^	0.44 ^a^	0.22 ^ab^	0.22 ^ab^	0.16 ^b^
	ether													
51	2-(2-ethoxyethoxy)-ethanol	MS RI	1617	1622	17.11 ^a^	4.76 ^b^	12.67 ^ab^	8.42 ^ab^	7.82 ^ab^	17.11 ^a^	7.65 ^b^	3.94 ^b^	4.17 ^b^	6.59 ^b^

^a^ Mode of identification: RI, retention index published in the literature data; MS, mass spectrum verified by comparison with mass spectra database (NIST 14.0). ^b^ Retention index. ^c^ Values bearing different lowercase letters within the same temperature are significantly different (*p* < 0.05).

## Data Availability

Data are contained within the article.

## Supplementary Materials

The following supporting information can be downloaded at: https://www.mdpi.com/article/10.3390/foods12234299/s1, Table S1: Sensory evaluation standard for stir-fried pork tenderloin.
