# Supplementary material for: Correlation Analysis between Volatile Compounds and Quality Attributes in Pork Tenderloin in Response to Different Stir-Frying Processes
Source: Foods. 2023 Nov 28;12(23):4299. doi: 10.3390/foods12234299 (PMC10706283; doi:10.3390/foods12234299)
Supplement: Supplementary file 1 [file foods-12-04299-s001.zip › foods-2703187-supplementary.pdf]

Table S1. Sensory evaluation standard for stir-fried pork tenderloin

Sensory attributes	Descriptors	Scores
color	golden	7-9
	yellow or light brown	4-6
	white or dark brown	1-3
texture	moderately tender	7-9
	medium tough or tender	4-6
	extremely tough or tender	1-3
aroma	rich aroma	7-9
	common aroma	4-6
	light aroma	1-3
overall flavor	good flavor	7-9
	general flavor	4-6
	poor flavor	1-3
